# Cerebral mechanism of opposing needling for managing acute pain after unilateral total knee arthroplasty: study protocol for a randomized, sham-controlled clinical trial

**DOI:** 10.1186/s13063-022-06066-6

**Published:** 2022-02-10

**Authors:** Chi Zhao, Hui Xu, Xinyu A, Bingxin Kang, Jun Xie, Jun Shen, Songtao Sun, Sheng Zhong, Chenxin Gao, Xirui Xu, Youlong Zhou, Lianbo Xiao

**Affiliations:** 1 Shanghai University of Traditional Chinese Medicine, Shanghai, 201203 China; 2 School of Acupuncture-Moxibustion and Tuina, Henan University of Chinese Medicine, Zhengzhou, 450003 China; 3 The First Affiliated Hospital of Henan University of Chinese Medicine, Zhengzhou, 450099 China; 4 Department of Joint Orthopaedics, Guanghua Hospital Shanghai University of Traditional Chinese Medicine, Shanghai, 200050 China; 5 Arthritis Institute of Integrated Traditional Chinese and Western Medicine, Shanghai Academy of Traditional Chinese Medicine, Shanghai University of Traditional Chinese Medicine, Shanghai, 200050 China; 6 Shanghai Guanghua Hospital of Integrated Traditional Chinese and Western Medicine, Shanghai, 200050 China

**Keywords:** Opposing needling, Functional magnetic resonance imaging, Acute pain, Total knee arthroplasty, Sham acupuncture, Central mechanism, Protocol

## Abstract

**Background:**

Opposing needling is a unique method used in acupuncture therapy to relieve pain, acting on the side contralateral to the pain. Although opposing needling has been used to treat pain in various diseases, it is not clear how opposing needling affects the activity of the central nervous system to relieve acute pain. We herein present the protocol for a randomized sham-controlled clinical trial aiming to explore the cerebral mechanism of opposing needling for managing acute pain after unilateral total knee arthroplasty (TKA).

**Methods:**

This is a randomized sham-controlled single-blind clinical trial. Patients will be allocated randomly to two parallel groups (A: opposing electroacupuncture group; B: sham opposing electroacupuncture group). The Yinlingquan (SP9), Yanglingquan (GB34), Futu (ST32), and Zusanli (ST36) acupoints will be used as the opposing needling sites in both groups. In group A, the healthy lower limbs will receive electroacupuncture, while in group B, the healthy lower limbs will receive sham electroacupuncture. At 72 h after unilateral TKA, patients in both groups will begin treatment once per day for 3 days. Functional magnetic resonance imaging will be performed on all patients before the intervention, after unilateral TKA, and at the end of the intervention to detect changes in brain activity. Changes in pressure pain thresholds will be used as the main outcome for the improvement of knee joint pain. Secondary outcome indicators will include the visual analogue scale (including pain during rest and activity) and a 4-m walking test. Surface electromyography, additional analgesia use, the self-rating anxiety scale, and the self-rating depression scale will be used as additional outcome indices.

**Discussion:**

The results will reveal the influence of opposing needling on cerebral activity in patients with acute pain after unilateral TKA and the possible relationship between cerebral activity changes and improvement of clinical variables, which may indicate the central mechanism of opposing needling in managing acute pain after unilateral TKA.

**Trial registration:**

Study on the brain central mechanism of opposing needling analgesia after total kneearthroplasty based on multimodal MRI ChiCTR2100042429. Registered on January 21, 2021

**Supplementary Information:**

The online version contains supplementary material available at 10.1186/s13063-022-06066-6.

## Background

Total knee arthroplasty (TKA) is one of the most commonly used methods for the treatment of advanced knee osteoarthritis (KOA) [1] as well as one of the most common elective surgeries in the USA [2]. It can effectively relieve pain, restore knee joint function, and improve quality of life, posture, and balance [3, 4]. Nevertheless, the potential postoperative complications following TKA, such as infection, pain, venous thrombosis, and fracture, are cause for concern for both surgeons and patients; therefore, it is necessary to control and reduce the incidence of these complications [5]. In the early stage of recovery after TKA, severe acute pain will occur [6], and inadequate pain management can delay the recovery process and may be accompanied by greater risks such as poor wound healing, prolonged hospitalization, unnecessary psychological stress, and reduced satisfaction. Severe postoperative pain also increases the risk of persistent long-term pain [7–9].

There are many ways to control postoperative pain [10]. “The panel recommends that clinicians offer multimodal analgesia…combined with nonpharmacological interventions, for the treatment of postoperative pain… (strong recommendation, high-quality evidence)” [10]. In clinical practice, surgeons and anesthesiologists generally utilize opioids for this purpose [11]. However, in orthopedic patients, preoperative opioids have been shown to result in preoperative hyperanalgesia and poorly controlled postoperative pain [12]. Furthermore, in patients undergoing primary arthroplasty, preoperative opioid use is a risk factor for increased postoperative narcotic consumption [13], postoperative complications [13–18], increased length of stay [15, 18], postoperative emergency room visits [14], revision surgery [14, 19, 20], readmission [13, 14, 18], increased costs [13, 18], and inferior clinical outcomes [21, 22].

Acupuncture, which is widely accepted and applied in daily life due to its exceptional therapeutic effects [23], has been used for several thousand years in China. It is a safe treatment method without severe side effects. As a non-pharmaceutical intervention, it not only can relieve early postoperative pain, but also helps to reduce the consumption of opioid analgesics [24]. Compared with other alternative therapies, acupuncture is safer and has greater potential for postoperative pain control [25]. Electroacupuncture (EA), a combination of traditional acupuncture and modern medicine that applies various levels of stimulating currents to acupoints via acupuncture needles, is relatively common in Chinese medical clinics and has a therapeutic effect on acute and chronic pain [26, 27]. Therefore, this method may be considered as an adjunct intervention for long-term pain relief in standard non-drug therapy [28]. Acupuncture to the knee joint on the operative side can relieve acute pain after TKA, but it may be associated with surgical incision infection [29]. In a previous study, to avoid this complication, we applied opposing needling (acupuncture to the unaffected knee) for pain management after TKA [28].

Opposing needling, an ancient nine-needle method, was first reported in Huangdi Neijing. Most Chinese medical practitioners use opposing needling therapy to treat unilateral limb pain [30]. This therapy has also been used in the field of functional rehabilitation and various types of neuralgia [31]. Studies have shown that contralateral and ipsilateral acupuncture have different analgesic regulation mechanisms and that contralateral acupuncture has a superior analgesic effect [32, 33]. This is mainly reflected in the different regulatory effects on the cingulate cortex. At present, there are more and more studies on the central mechanism of acupuncture analgesia by using neuroimaging, mainly focusing on chronic pain such as migraine, low back pain, neck pain, and so on. In the field of acupuncture treatment of postoperative acute pain, there are few studies on the brain function mechanism. Although we have previously applied opposing needling in a pain management program after TKA, the specific brain mechanisms underlying this therapy remain unclear.

### Objectives

The current study protocol aims to (1) investigate the influence of opposing EA treatment on the brain activities of patients after unilateral TKA compared with that of sham opposing EA treatment and (2) analyze the possible correlations between the changes in cerebral activity and the improvement of clinical variables in each group, exploring how opposing EA modulates brain function to manage acute pain after TKA.

## Methods/design

### Study design and participants

This clinical neuroimaging study aims to investigate the central mechanism by which opposing EA alleviates acute knee pain after TKA. The participants will be recruited from the outpatient or inpatient department of joint surgery in Shanghai Guanghua Hospital of Integrated Traditional Chinese and Western Medicine, Shanghai, China, which is a teaching and tertiary hospital of Shanghai University of Traditional Chinese Medicine, Shanghai, China. The participants will comprise patients with osteoarthritis who require TKA (excluding surgical contraindications). After admission, the patients will undergo initial right TKA as planned. The day before the operation, a baseline assessment of eligible patients will be conducted, the participants will receive a thorough explanation of the study aim and protocol, they will be told that the study will not involve collecting biological samples for storage, and written informed consent will be obtained. All participants will be informed of the random distribution of opposing EA and sham opposing EA treatments, as well as the possible risks. They will be free to withdraw from the study at any time without any fines or loss of benefits. However, we will attempt to understand the reasons for withdrawal and will encourage participants to participate in the study as much as possible. We followed the SPIRIT 2013 Guidelines [34] and STRICTA [35] to report the trial protocol. This study has been approved by the ethics committee of Shanghai Guanghua Hospital of Integrated Traditional Chinese and Western Medicine (Ethics Approval Number: 2020-K-103) and registered in the Chinese Clinical Trial Registry.

Nine investigators will be involved in this study, including a chief orthopedic physician (LBX) with 20 years of clinical experience, two orthopedic surgeons (JX and STS), two Chinese medicine acupuncturists (BXK and CZ), and two outcome evaluators (CXG and SZ). XYA and XRX will recruit hospital patients who meet the inclusion criteria and will introduce the trial process, possible benefits, and risks to patients to obtain their informed consent.

### Inclusion criteria

Subjects who meet the following inclusion criteria are eligible to participate: (1) aged 40–70 years and right-handed; (2) meet the Clinical Classification Criteria for Osteoarthritis of the Knee as recommended by the American College of Rheumatology; (3) scheduled to undergo initial right TKA under general anesthesia without contraindications; (4) undergone similar surgical approach; (5) American Association of Anesthesiologists Grade I or II [36]; (6) posterior cruciate-stabilizing prostheses (Smith & Nephew, London, UK); (7) postoperative X-ray shows good alignment of knee prosthesis; and (8) consciousness is clear and cognitive function is normal.

### Exclusion criteria

Patients will be excluded if they (1) abuse alcohol, drugs, or other medications that may influence brain imaging outcomes; (2) have psychiatric, neurologic, gastrointestinal, cardiovascular, infectious, immunologic, respiratory, or renal illnesses; (3) have any other chronic pain conditions or a history of head trauma with loss of consciousness; (4) have a diagnosis of rheumatoid arthritis or other leg-related pain disorders; (5) have magnetic resonance imaging (MRI) contraindications such as claustrophobia, a cardiac pacemaker, a defibrillator, heart stenting, or an intrauterine device; or (6) skin damage in the acupoint area.

### Exit criteria and management

Exit criteria include (1) participants request the suspension of the trial process, (2) severe postoperative complications (e.g., pulmonary embolism, infection), and (3) intra-treatment side effects. Participants who drop up during the trial will be treated with routine analgesia.

### Sample size

The sample size estimation for neuroimaging is different from that of classical randomized controlled trials. Compared with the evaluation of curative effect, neuroimaging studies focus more on mechanisms of the effects. Previous literature indicates that, for MRI studies, 12–15 participants in each group are sufficient [37, 38]. To obtain stable results of brain functional network analysis, the current study design requires at least 20 participants in each group. However, considering a 20% drop-out rate during the trial and the possibility of head movement artifact during the MRI scan that may render the images useless, we plan to recruit 80 participants (40 patients per group). Each group will undergo two MRI scans to investigate the different central responses between contralateral acupuncture and sham acupuncture after right TKA.

### Recruitment strategies

**Fig. 1:**
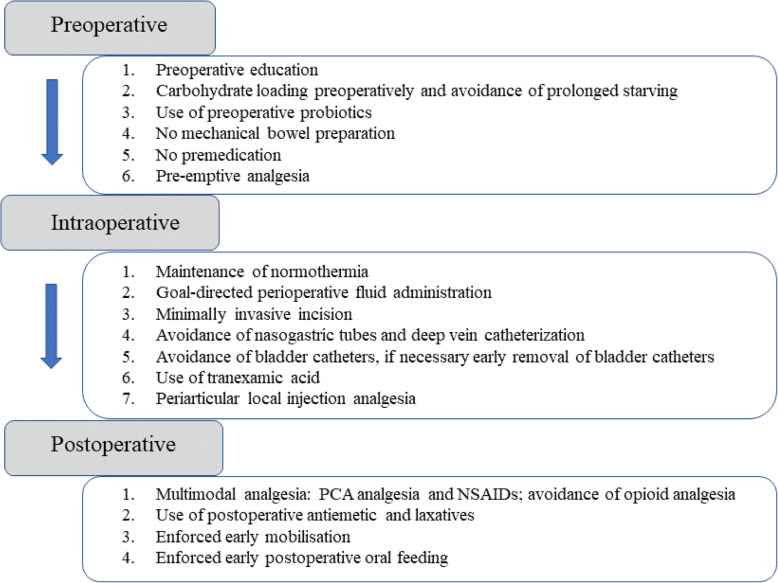
Enhanced recovery after surgery

**Fig. 2:**
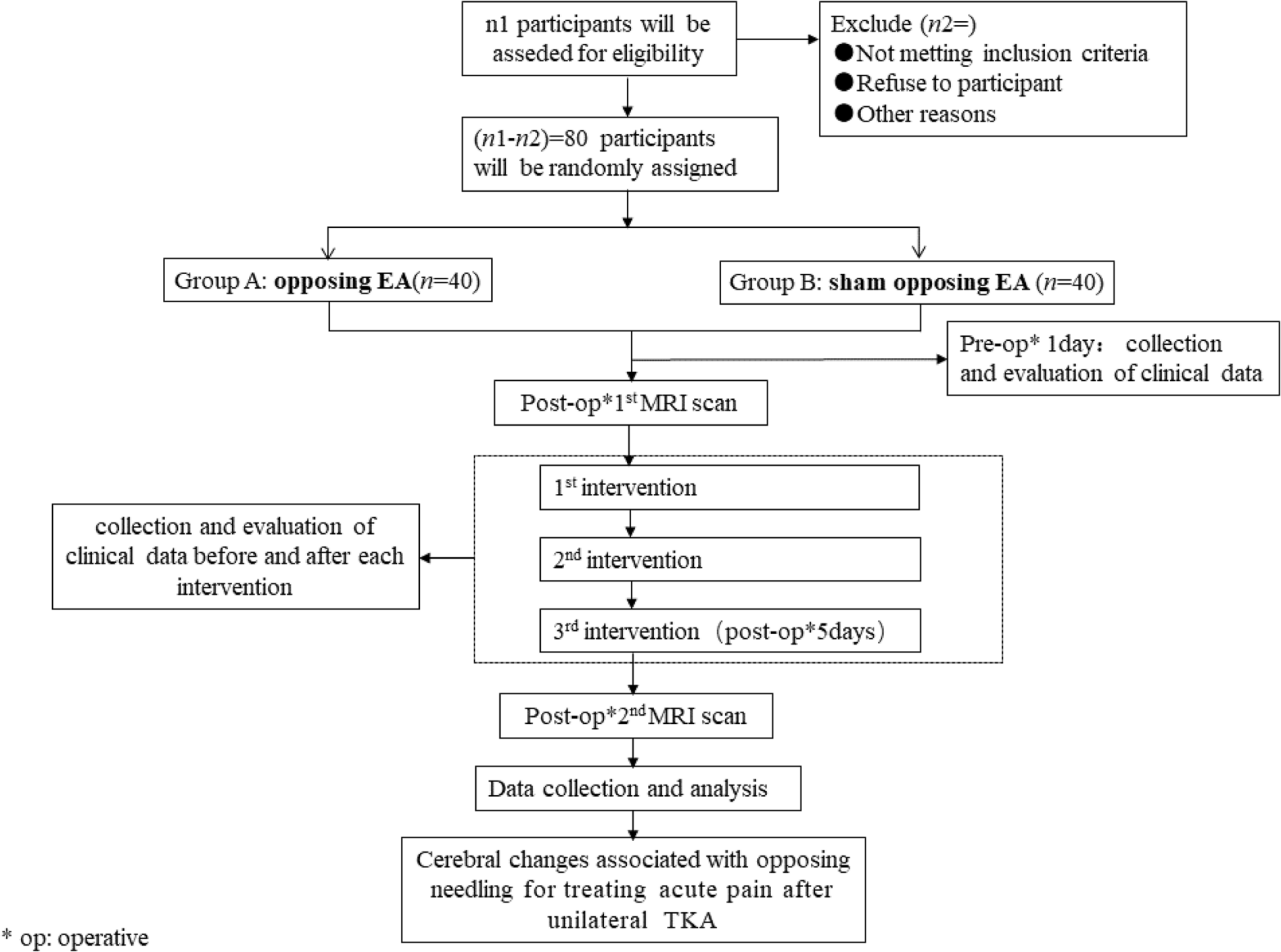
Flow diagram of the trial. EA, electroacupuncture; op, operative; TKA, total knee arthroplasty; MRI, magnetic resonance imaging

**Fig. 3:**
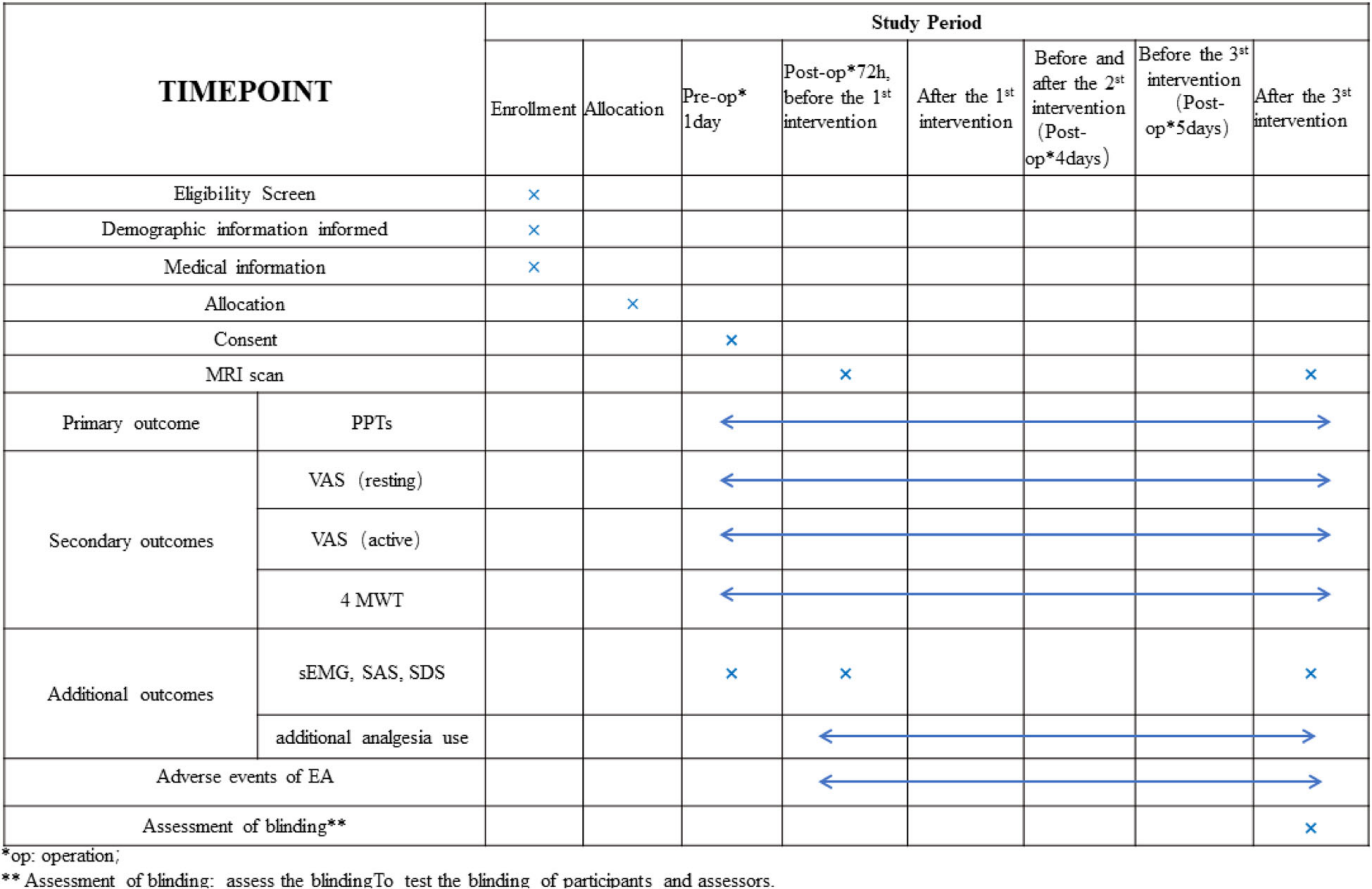
Schedule of trial enrollment, interventions, and assessments

Participant registration will be conducted between March 2021 and January 2022. In order to include any other scientific publications, we will obtain written informed consent from all participants. When the participant is hospitalized, a trained nurse and several physiotherapists will implement the enhanced recovery after surgery approach (Fig. 1). Figure 2 presents the trial flow, including participant recruitment, eligibility screening, randomization, intervention, and outcome assessments. Figure 3 presents an overview of the trial design, conduct, review, and analysis. A completed SPIRIT 2013 checklist (Word) is presented in Additional file 1.

### Randomization and blinding

An independent researcher will use statistical software SPSS 25.0 to ensure random grouping. Eligible and consenting patients with KOA who have undergone initial right TKA will be randomly assigned to the opposing EA group or the sham opposing EA group at a ratio of 1:1 (each group, *n* = 40). Random grouping steps: (1) setting random seeds, (2) generating random numbers (set range 0–1), (3) sorting grouping, and (4) obtaining random grouping results. Concealed grouping will be carried out using continuously encoded opaque envelopes containing treatment information. Only the acupuncture therapist in charge of EA treatment will be permitted to open the envelope.

**Fig. 4:**
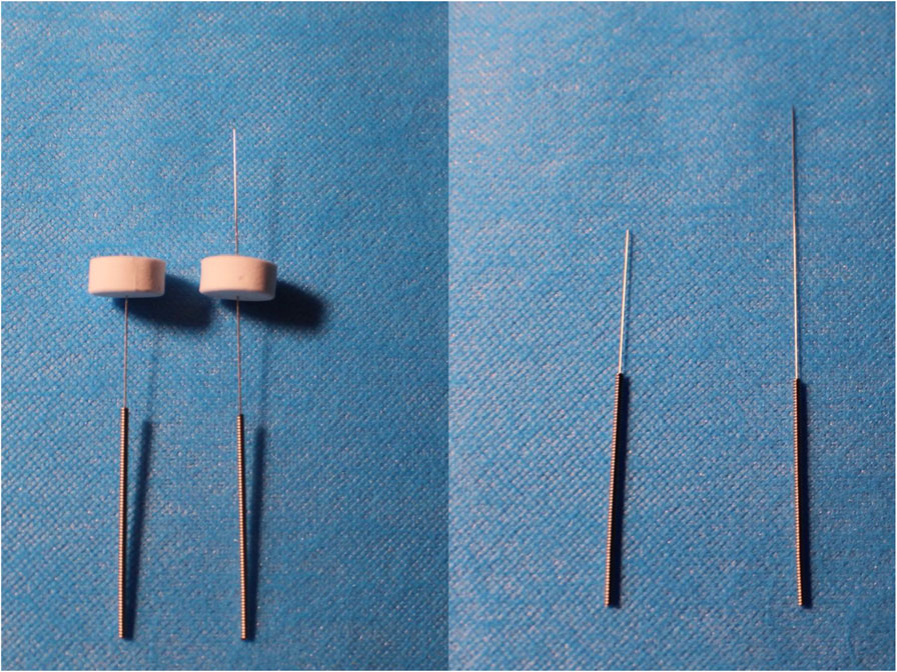
Difference in the needles and acupuncture depth between the electroacupuncture and sham electroacupuncture groups. The tip of the pragmatic placebo needle (left) is blunt. The needle will be inserted into a sterile adhesive pad without puncturing the skin. The regular acupuncture needles (right) will be inserted into the skin of the corresponding acupoints through a sterile adhesive

All participants, physiotherapists, orthopedic surgeons, result evaluators, and data statisticians will be unaware of the trial grouping to reduce the risk of bias. To maximize the blinding of patients, an effective sham opposing EA and a pragmatic placebo needle design will be applied with adhesive pads, a dull-tipped placebo needle, and a sham electrical stimulation device (Fig. 4).

### Patient safety

The study will be conducted in accordance with the guidelines and principles of the Declaration of Helsinki. Adverse events caused by acupuncture, such as subcutaneous hemorrhage, dizziness, or other severe events, will be dealt with immediately and recorded in detail in the case report form. Each patient will be compensated to participate in the study.

### Interventions and analgesia

#### Basic physical therapy

Both groups will begin basic physiotherapy (exercise) 24 h after TKA. This will include hip flexion (straight leg elevation), knee flexion extension, painless knee loosening, and ankle pumping exercises. The treatment period will be supervised by a physiotherapist, and the program will last for 30 min per day for 2 weeks. In addition, participants must perform breathing exercises with lower limb elevation within 24–48 h after TKA.

#### Scheme of standard analgesic use

Within 48 h after TKA, participants will use a patient-controlled analgesia pump. Fentanyl (Yichang Renfu Pharmaceutical Co., Ltd., Yichang, China) will be used as an analgesic drug at a continuous infusion rate of 0.25 μg/kg/h, a bolus dose of 0.15 μg/kg, and a 10-min lockout time. In addition, all participants will begin oral administration of celecoxib capsules (Pfizer Pharmaceutical Co., Ltd., New York, USA), at a dosage of 200 mg once daily, 72 h after operation.

#### Opposing EA

**Fig. 5:**
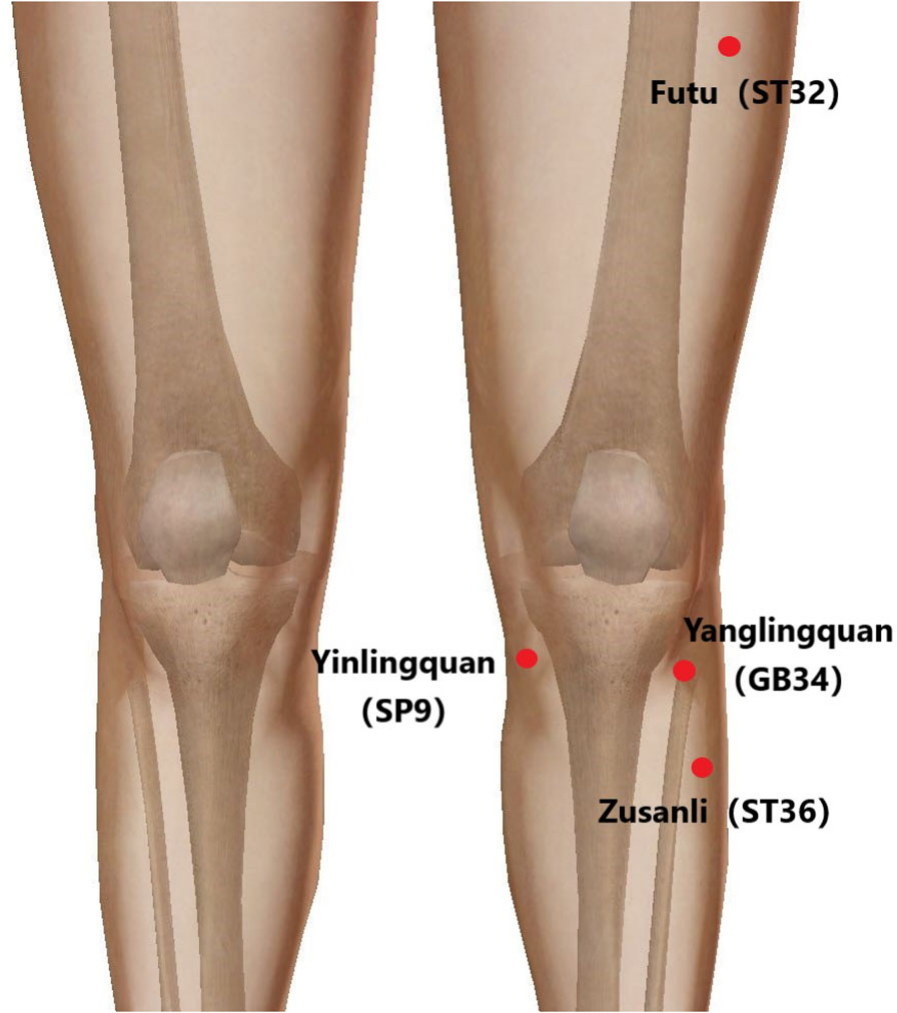
Location of acupoints for the opposing electroacupuncture and sham opposing electroacupuncture groups

According to the new standard of “International Acupuncture Nomenclature” [39], the acupoints of *Yinlingquan* (Spleen 9, SP9), *Yanglingquan* (Gallbladder 34, GB34), *Futu* (Stomach 32, ST32), and *Zusanli* (Stomach, ST36) will be applied to the healthy lower limb (contralateral side) (Fig. 5). After skin disinfection, a sterile rubber pad (8 × 10 mm, Suzhou Medical Supplies Factory, China) will be placed on the above acupoints, and the acupuncture needle (0.25 × 40 mm, Suzhou Medical Supplies Factory, China) will pierce the skin at a depth of approximately 3 cm. The acupuncturist will use the same acupuncture technique to produce feelings of *de qi* (patient sensations of acidity, sinking, distension, and fever at the acupoints). After achieving *de qi* sensation, the Huatuo SDZ-II EA apparatus (Suzhou Medical Supplies Factory, Suzhou, China) will be used to continuously stimulate the acupoints. The frequency of the instrument will be adjusted to 40 Hz, and the pulse width will be adjusted to a constant current square wave of 1 ms. It is appropriate to gradually increase the pulse intensity until it becomes unbearable. The opposing EA group will undergo a 3-day treatment course consisting of 30-min sessions once daily.

#### Sham opposing EA

In the sham opposing EA group, a pragmatic placebo needle will be used [28]. As in the opposing EA group, the *Yinlingquan* (Spleen 9, SP9), *Yanglingquan* (Gallbladder 34, GB34), *Futu* (Stomach 32, ST32), and *Zusanli* (Stomach, ST36) acupoints will be selected. The blunt needle (size 0.25 × 30 mm, Hwato Brand, Suzhou Medical Appliance Factory, China) used at these acupoints will not penetrate the skin but will affix to the surface of the skin through an adhesive pad (Fig. 5). The same acupuncture manipulation as that of the opposing EA group will be used to simulate the genuine acupuncture process. A Huatuo SDZ-II EA apparatus (Suzhou Medical Supplies Factory, Suzhou, China) will be connected to these acupoints through special electrode wires without electric output. The sham needle will remain in the adhesive pad for 30 min during each treatment session. The process of sham opposing EA is similar to that of opposing EA, but the former does not involve skin penetration, needle manipulation, or electrical output. The sham opposing EA group will also undergo a 3-day treatment course consisting of 30-min sessions once daily. Both groups will be treated by experienced acupuncturists with standardized training.

To ensure treatment compliance, participants will be required to register for treatment. Throughout the trial, the participants will be treated separately to prevent communication between participants. Routine care pathways (including the use of any drugs) will be carried out as standard and will not be affected by the implementation of EA or sham EA. Participants who completed the trial will receive free elastic band and rehabilitation ball to assist in the rehabilitation of lower limb muscles. In addition, we will give them a 6-month follow-up and postoperative rehabilitation exercise guidance.

### MRI data acquisition

The functional MRI (fMRI) data will be acquired by a clinical 1.5-T whole-body MRI scanner (United Imaging, Shanghai, China) at the MRI Center in Guanghua Hospital, Shanghai University of Traditional Chinese Medicine. The participants will be asked to wear soundproof earplugs during the scan, and the examiner at the MRI center will add a sponge between the patient’s head and the head coil to reduce noise and to discourage patient head movement. The participants will be instructed to stay awake and relaxed and to close their eyes during the scan and not think of anything in particular. Each patient will undergo two MRI scans; the first scan will be performed before the first intervention after surgery, and the second after all interventions are completed. After each scan, participants will complete a simple questionnaire to determine if they fell asleep during the scan.

Resting-state fMRI (rs-fMRI) images will be generated using a rapid-gradient echo-planar imaging sequence with the following settings: repetition time, 3,000 ms; echo time, 30 ms; flip angle, 90°; field of view, 225 × 225 mm^2^; acquisition matrix, 64 × 64; 43 slices; slice thickness 3.5 mm; voxel size, 3.52 × 3.52 × 3.52 mm^3^; and bandwidth, 2250 Hz/pixel. Each scan will require 12 min 13 s. Three-dimensional T1-weighted magnetization-prepared rapid-gradient echo sagittal images will be captured using the following parameters: repetition time, 10.4 ms; echo time, 4.4 ms; inversion time, 750 ms; flip angle, 10°; matrix, 256 × 232; slice thickness, 1 mm; and voxel size, 1 × 1 × 1 mm^3^. Each scan will require 3 min 32 s.

### Outcome evaluations

The clinical evaluations will measure knee pain and physical function. The primary outcome measure will consist of the pressure pain thresholds (PPTs), and the secondary outcome indicators will include the visual analogue scale (VAS), including pain during rest and activity, and the 4-m walking test (4MWT). Additional outcome indicators will comprise surface electromyography (sEMG), the self-rating anxiety scale (SAS), the self-rating depression scale (SDS), and the use of additional analgesics. Data regarding PPTs, VAS, and the 4MWT will be collected at baseline, 30 min before each acupuncture treatment, and immediately after each acupuncture treatment. sEMG, SAS, and SDS data will be collected at baseline, before the first acupuncture session after the operation, and after all interventions have been completed. The use of additional analgesics will be assessed at the end of the intervention period.

#### Primary outcome

PPTs are quantitative sensory tests that are widely used in the prediction and evaluation of clinical therapeutic effects in patients with KOA [40–43]. An experienced trained clinician (CXG) will perform all PPT measurements using the FDX-50 portable pain tester (Wagner Instruments, Greenwich, CT, USA) to minimize measurement variability. The specific operation is as follows: the patient will lie down in the supine position, and two points of the operated knee joint will be used for testing: (1) lateral point: located 3 cm lateral to the midpoint of the lateral edge of the patella, and (2) medial point: located 3 cm medial to the midpoint of the medial edge of the patella. A 1-cm^2^ probe will be applied perpendicular to the skin, and the pressure will be maintained at 10 kPa/s. Patients will be instructed to say “stop” the first time they feel pain, at which point the PPT readings will be recorded. Three readings will be taken at each site, with the final two readings averaged to increase the reliability. There will be a small change in the probe position of the pain meter on the skin between each reading to avoid sensitization of the test area [44].

#### Secondary outcomes

The VAS score will be used to measure pain. Postoperative pain assessment should evaluate pain at rest and with movement [45]. Independent evaluators who do not participate in acupuncture will perform the VAS assessments (participants will be asked to indicate the intensity of pain at the most painful point while resting and walking). In this experiment, a line segment of 10 cm will be used as a scale, and the ends will be marked as 0 (painless) and 100 (severe). Patients will draw a vertical mark and horizontal line crossing to evaluate their own pain and the distance from 0 to the marked point will be measured to determine the VAS score; the higher the score, the higher the degree of pain [46]. Throughout the trial, in addition to evaluating resting pain at 6 h after the operation, pain will be evaluated before and after each intervention after the operation.

The 4MWT can be used as a supplementary index to evaluate lower limb function after orthopedic surgery, and the test-retest reliability is good [47]. We will use a 50-m corridor in the orthopedic ward for evaluation because this test requires additional distances to accelerate and decelerate. We will mark the 8-m path needed for the 4MWT in the corridor; the first 2 m of this path will be marked with an acceleration warning, and the final 2 m will be marked with a deceleration warning. These extra spaces for acceleration and deceleration will not be used as data collection areas. The starting and end points of the 4-m path will be marked with colored inelastic tape. When the patient’s leg crosses the acceleration starting point, the clock starts. Once the patient’s leg passes the 4 m mark, the stopwatch stops, signaling the patient to slow down and stop. Before the test, we will provide verbal guidance to the patients, explain the detailed test procedures, and tell patients to walk as soon as possible. All patients will use walkers as aids to ensure safety [47].

#### Additional outcomes

SEMG is widely used in the field of clinical neurophysiology and can be used not only for functional diagnosis, but also for monitoring treatment results. Currently, orthopedic injury is one of the most prominent application fields [48–50]. Before sEMG signal acquisition, participants will be told to wear loose trousers to expose the thighs at a room temperature of approximately 25 °C. To ensure the quality of the EMG signals collected, the evaluator should shave the hair from the leg area where the electrode is attached and disinfect it with 75% alcohol before attaching the electrode. The placement requirements for disposable ECG electrodes (Ag/Cl, T-800) include (1) the central distance of the bipolar electrode is 2 cm; (2) the central connection of the bipolar electrode is parallel to the long axis of the muscle fibers below it; and (3) the bipolar electrode should be attached to the ventral portion of the medial vastus muscle, rectus femoris muscle, and lateral vastus muscle. The belly of the rectus femoris is located at the midpoint of the line between the anterior superior iliac spine and the superior edge of the patella, and the belly of the lateral femoral muscle is located at the midpoint between the greater trochanter and the inferior edge of the patella. Because preoperative and postoperative pain leads to an inhibitory protective reflex of the medial vastus muscle and disuse atrophy of some muscle fibers, it is difficult to identify the belly of the medial vastus muscle quickly and accurately. The evaluator will place a towel roll under the healthy popliteal fossa and ask the participant to press the towel roll into the bed as forcefully as possible to straighten the knee joint. At this point, there is a visible protuberant area above the medial patella, that is, the belly of the medial vastus muscle on the operative side relative to the healthy side; thus, the specific position of the belly of the medial vastus muscle on the operative side can be determined; (4) the reference electrode will be placed at the fibular head. The sEMG signals of the quadriceps femoris will be measured and recorded using the TeleMyo 2400 wired sEMG tester (NORAXON USA Inc., Scottsdale, AZ, USA) at a sampling frequency of 1500 Hz. Channel 1 connects with the right lateral vastus muscle, channel 2 connects with the left medial vastus muscle, channel 3 connects with the right rectus femoris muscle, channel 4 connects with the left rectus femoris muscle, channel 5 connects with the right medial vastus muscle, and channel 6 connects with the left lateral vastus muscle. To reduce the interference to the EMG signal caused by the small movement between the metal chuck and the electrode piece of the EMG acquisition line, disposable medical rubber paste will be used to fix the EMG acquisition line metal chuck and the electrode piece. If it is too tight, fixation will be poor; therefore, the fixed tightness of the plaster is adjusted with the minimum influence on the zero EMG value as a reference. Simultaneously, under the premise of not affecting the exercise mode, the EMG acquisition line should be fixed to prevent pulling in the processes of sitting and standing up.

When conventional analgesia programs cannot effectively improve pain, additional analgesics will comprise intramuscular injection of 40 mg parecoxib sodium (Pfizer Pharmaceutical Co., Ltd., national drug standard: J20130044). The frequency and time of use will be recorded.

Furthermore, to study the effect of emotional state on brain activity, the SAS and SDS will be applied. The SAS and SDS have high validity and objectivity in clinical research; they are often used not only to screen for depression, but also as indicators of severity and measurement of outcome. For example, in the study of chronic pain management, they are used as one of the observation indicators [51]. Patient emotional state is evaluated by scoring 20 related items in each of the two scales [52, 53].

### Data management and monitoring

Clinical data will be carefully preserved using paper and electronic case report forms. It is stipulated that only result evaluators have access to case report forms, and they will check the data entered to ensure no duplication. In the course of the trial, Shanghai Guanghua Integrated Traditional Chinese and Western Medicine Hospital will conduct regular reviews, and the hospital ethics committee will supervise every week to avoid violations. The final experimental dataset will not be accessible to anyone except specialized data statisticians, and the dataset will contain only encoded data and will not contain participant information. The relevant research team will monitor the safety, progress, research integrity, and design aspects at several meetings. The Finance Section of the Shanghai Guanghua Integrated Traditional Chinese and Western Medicine Hospital will audit the costs of this study.

### Adverse events of EA

The main EA-related complications include dizziness, acupuncture stagnation, acupoint hematoma, pain > 2 h after continuous acupuncture, and bleeding. Any such adverse events during the trial will be recorded.

### Blinding assessment

To evaluate blinding, we will use a questionnaire completed within 5 min of the final intervention. The percentage of participants from each group who believed they had received real EA treatment will be recorded. The difference in the success rate of blinding among the participants will be determined.

### Statistical analysis

Based on the intention-to-treat principle, a full analysis set and per-protocol sets will be used for the primary analysis. Sensitivity analysis will be performed to determine the impact of incomplete records on the results. Missing data will not be imputed. For the clinical data, statistical analyses will be performed using the statistical package IBM SPSS Statistics V21 (IBM Corp., Armonk, NY, USA). Complete follow-up assessments of the participants will be performed using the per-protocol and intention-to-treat populations. The statistical analysis will include four aspects. First, for the measured data with normal and non-normal distributions, the mean ± SD and median will be used to describe the concentration trend and dispersion degree, respectively. Two independent sample *t*-tests and Mann-Whitney *U* tests will be used to compare the differences between groups. Second, the quadruple chi-squared test will be used to evaluate the sex composition ratio (which is a classified variable). Third, the Mann-Whitney *U* test will be used to compare the differences between the groups in the ranked data. Finally, a generalized linear mixed model will be used to compare the repeated measurement data. The statistical significance will be set to two-sided *p* < 0.05.

The fMRI scan data will be preprocessed and analyzed using SPM12 software (http://www.fil.ion.ucl.ac.uk/spm/) performed on MATLAB 2015b (MathWorks, Inc., Natick, MA, USA). The main preprocessing steps are as follows: (1) remove the functional image data of the first 10 time points while waiting for the imaging gradient to reach a stable state; (2) carry out time correction, remove the time difference between different layers by correction, and then perform head correction; (3) match the functional image to the corresponding anatomical image and convert it to a standard Montreal Neurological Institute space—the resampling size of the image data pixels is 3 × 3 × 3 mm^3^; (4) use the linear regression method to remove the related variables, including the average signal of the cerebrospinal fluid and 12 cephalic parameters; and (5) process the data by removing high-frequency noise and low-frequency drift. The data are mainly processed by linear drift and band-pass filtering (0.01–0.08 Hz). After data preprocessing, the amplitude of low-frequency fluctuation, regional homogeneity, and functional connectivity will be used to investigate the cerebral responses of the different study groups. A two-sample *t*-test will be used to evaluate intra- and inter-group differences to analyze possible brain responses, and a correlation analysis will be conducted to investigate the changes in fMRI image data and the corresponding clinical data in each group.

## Discussion

This is a randomized, sham-controlled fMRI study to explore how opposing needling acupuncture relieves postoperative knee pain by regulating brain function after TKA in patients with KOA.

In recent years, pain management has been widely used in the perioperative period as a core concept of accelerated rehabilitation surgery [54, 55]. Optimal postoperative pain management can effectively prevent and reduce adverse reactions and stress reactions. For this, multimodal analgesia should be adopted, and the development of multidisciplinary analgesia methods should be promoted [56, 57]. Acupuncture, a traditional Chinese medicine, has achieved good results in promoting analgesia after TKA [58, 59].

According to the traditional acupuncture meridian theory and the principle of acupoint adjacent treatment, SP9 and GB34 near the knee joint have been selected as the acupoints of the foot Taiyin spleen meridian and foot Shaoyang gallbladder meridian, respectively, for use in this study. In addition, we select the ST36 and ST32 acupoints of the stomach meridian of foot Yangming. In the theory of traditional Chinese medicine, spleen *qi* is the biochemical source of human *qi* and blood; therefore, these two acupoints have been selected to promote the operation of human *qi* and blood and to improve the state of *qi* and blood stasis caused by injury to the knee joint after surgery.

Opposing needling refers to acupuncture to healthy lower limbs. Increasing evidence from clinical and experimental studies shows that opposing needling has a protective effect and promotes functional rehabilitation, but its mechanism remains to be elucidated [60]. In patients who have undergone orthopedic lower limb surgery, acupuncture on the unaffected limb is more acceptable, and patient compliance is higher. Furthermore, this acupuncture method avoids direct invasive intervention in the surgical area and reduces the risk of postoperative infection to a certain extent.

FMRI is a large-scale neural network technique. As the main type of fMRI, blood oxygen level-dependent fMRI measures neuronal activity using the ratio of oxyhemoglobin to deoxyhemoglobin as a contrast mechanism [61]. With the advantages of high imaging speed, non-radiation, and non-invasiveness [62, 63], it has become a commonly used neuroimaging technique in the research of the central mechanism of acupuncture [64, 65]. Relative to task-related fMRI, rs-fMRI has the advantage of providing more comprehensive information on the functional architecture of the brain [61]. Using rs-fMRI, some investigators have found that acupuncture can exert an analgesic effect by directly regulating the anterior cingulate gyrus. Their results also suggested that acupuncture on the opposite side and ipsilateral side can induce different brain mechanistic changes [33].

In this trial, we will adopt direct quality control measures to reduce bias and improve the reliability of the results. In addition to establishing strict inclusion and exclusion criteria at the stage of participant recruitment, demographic characteristics such as age and handedness will also be applied as inclusion criteria. The mode of operation and the type and dose of perioperative medication will be regulated as much as possible, and factors that may affect brain activity, including coffee drinking or alcohol and drug abuse, will also be considered. The acupuncturist will receive standardized training before the trial and carry out all treatments in accordance with standard operating procedures. During acupuncture, unnecessary communication between the acupuncturist and the patient will be prohibited. The personnel responsible for the analysis will be trained and blinded to the test group.

In summary, the purpose of this trial is to study the main mechanism of opposing EA in the management of acute pain after TKA by analyzing the correlation between changes in brain activity and clinical variables. We hope that our findings can provide a visual reference for the clinical application of opposing needling after TKA.

## Trial status

The trial is currently in the process of recruiting participants. Recruitment for the trial began on March 10, 2021, and is expected to be completed on March 31, 2022.

## Supplementary Information

**Additional file 1:.** SPIRIT 2013 Checklist

## Data Availability

The datasets and the informed consent forms can be obtained from the corresponding author upon reasonable request.

## Abbreviations

TKA: Total knee arthroplasty
KOA: Knee osteoarthritis
EA: Electroacupuncture
MRI: Magnetic resonance imaging
fMRI: Functional magnetic resonance imaging
rs-fMRI: Resting-state functional magnetic resonance imaging
PPTs: Pressure pain thresholds
VAS: Visual analogue scale
4MWT: 4-m walking test
sEMG: Surface electromyography
SAS: Self-rating anxiety scale
SDS: Self-rating depression scale
